# Evaluating eHealth Interventions: The Need for Continuous Systemic Evaluation

**DOI:** 10.1371/journal.pmed.1000126

**Published:** 2009-08-18

**Authors:** Lorraine Catwell, Aziz Sheikh

**Affiliations:** Centre for Population Health Sciences, The University of Edinburgh, Edinburgh, Scotland

## Abstract

In the first in a series of three articles on evaluating eHealth, Aziz Sheikh and Lorraine Catwell outline the background to the series and discuss the importance of evaluating the widespread investments in and adoption of information communication technology in health care.

*This is the first in a monthly series of three articles on evaluating eHealth.*

**Summary Points**

- eHealth interventions will play a substantial role in shaping health care systems in the 21st century.
- Until eHealth interventions are “fit for purpose”, health care professionals are unlikely to adopt them and this risks implementation failure.
- eHealth developments should be viewed as interventions, and evaluated as new drugs or management programmes, recognising the challenges of evaluating complex interventions.
- We propose a means to evaluate eHealth interventions while they are being designed, developed, and deployed.
- We argue that continuous systematic evaluations of eHealth interventions are needed.

There is now considerable interest internationally in exploiting the potential of information communication technology (ICT) systems to improve the quality, safety, and efficiency of health care. Given that the adoption of ICT systems by health care providers is some 25 to 30 years behind many other private and public sectors [1], there is an understandable sense of urgency with which these eHealth initiatives are now being commissioned, developed, and deployed, typically at considerable expense [2]–[5]. The American Recovery and Reinvestment Act of 2009 [6], which includes $34 billion to incentivise health care professionals to “use a certified EHR (electronic health record) technology in a ‘meaningful manner’,” is a recent high-profile example of the sums of money that are being invested in eHealth [7].

Whilst eHealth interventions undoubtedly have the potential to play a substantial role in shaping and helping to create health care systems that are fit for the 21st century [8], experience has repeatedly shown that if attempts are made to implement poorly designed systems, there is a real danger that not only will the anticipated benefits fail to be realised [4],[5],[8]–[10], but also that vast sums of money will have been squandered in the process. Worse still, patients' safety may also be compromised [11]–[13].

The argument about the need rigorously to evaluate medical technologies themselves as well as their social and economic impact is not new [14]–[16]. However, those responsible for ICT developments must appreciate that health information systems should be evaluated with the same rigour as a new drug or treatment programme, otherwise decisions about future deployments of ICT in the health sector may be determined by social, economic, and/or political circumstances, rather than by robust scientific evidence.

Health care is in what Heathfield has described as “a catch 22 situation” [17]: until we develop eHealth interventions that are “fit for purpose” [18]—and early evaluations suggest they are frequently not [19]—health care professionals are, justifiably, reluctant to adopt these new technologies. The paradox is that unless we have the means to demonstrate the true benefits of these systems, which requires integrating these technologies throughout the health care industry, we will never have the necessary evidence to support the case for ICT in health care [17].

So, while financial incentives to adopt certain eHealth interventions may be understandable [6],[7], they should never be the main reason for their adoption. Instead, society must be able to judge the true value of eHealth interventions in its own right. Therefore a means simultaneously to evaluate eHealth interventions while they are being developed and deployed is required [20]. In this article, we argue for continuous systematic multifaceted evaluations—throughout the lifecycle of eHealth interventions—on the grounds that such an evaluative approach is likely to provide timely and relevant insights that can help to assess the short-, medium-, and long-term safety, effectiveness, and cost-effectiveness of eHealth interventions. The suggested lifecycle–based approach to evaluation should [20], we believe, become the norm rather than the exception, as is currently the case.

## What Is eHealth?

In the past, the term “medical technology” was often used to describe the set of techniques, drugs, equipment, and procedures used by health care professionals to deliver medical care to individuals. ICT deployments would historically therefore have been considered under this heading [14]. Today, eHealth is the term more commonly used in relation to ICT deployments in health care; although there have been several attempts to define eHealth (see Box 1 for some examples), there is still no universal agreement on the precise meaning of this term.

**Box 1. Example definitions of eHealth**

- “…a consumer-centred model of health care where stakeholders collaborate, utilising ICTs including Internet technologies to manage health, arrange, deliver and account for care and manage health care systems” [4].
- “… today's tools for substantial productivity gains, while providing tomorrow's instrument for restructured, citizen-centred health systems” [10].
- “… describes the application of information and communications technologies (ICT) across the whole range of functions that affect healthcare, from diagnosis to follow-up. It is the means to deliver responsive healthcare tailored to the needs of the citizen” [21].

Consider, for example, telemedicine interventions, i.e., the provision of health care services across distances by such means as telemonitoring devices (e.g., teleradiology and telecardiology), teleconsulting, or even telesurgery [21]–[24]. Numerous electronic medical records systems are used, for example, to record details of patients' health and their medication [25],[26]. Finally there are the many health portals that provide a means to access medical records and health-related information over a secure network, such as Google Health, Microsoft HealthVault, and the National Health Service's HealthSpace [27]–[29].

All of these systems have been defined as eHealth technologies [4],[10],[21], but all may serve very different purposes and all may have very different target user groups. For this reason, we suggest that any definition of eHealth should encompass the full spectrum of ICTs, whilst appreciating the context of use and the value they can bring to society. One such definition that includes these various facets is that proposed by Pagliari [30], who defined eHealth as:

*“…an emerging field of medical informatics, referring to the organisation and delivery of health services and information using the Internet and related technologies. In a broader sense, the term characterises not only a technical development, but also a new way of working, an attitude, and a commitment for networked, global thinking, to improve healthcare locally, regionally and worldwide by using information and communication technology.”*

## The Anticipated Benefits of eHealth

As has been the case in many other sectors, it is widely believed the introduction of ICT systems within health care, combined with the necessary social (i.e., organisational and behavioural) changes [31], will substantially reduce costs and improve efficiency [10]; it is also anticipated that eHealth will lead to a reduction in the high number of patients who are inadvertently harmed by medical errors and violations [12].

For example, proponents of the introduction of electronic health records, which are currently being introduced in England, Scotland, France, Canada, Australia, and the USA, anticipate that such tools will lead to improvements in the recording, storing, retrieving, and sharing of patient information both within and between various stakeholder groups. Their hope is that this will, in turn, translate into improvements in the delivery of health and social care [26].

Similarly, telemedicine initiatives [21]–[24], which increasingly are being deployed in the context of the management of people with long-term conditions, have the ability to transcend many of the challenges health care professionals face in providing equitable, accessible, and high quality care to people living in remote locations and/or those who are housebound [4],[10]; such interventions can also improve the convenience of care by delivering it to people in the comfort of their own homes [4],[10]. Given that health care systems are (directly or indirectly) a major emitter of carbon gasses, the widespread use of telemedicine could also result in ecological benefits.

Another important initiative that offers considerable potential benefit is the development of health portals, which provide health care professionals, patients, their families, caregivers, and the public at large with direct access to health records as well as to relevant and accurate medical information at the touch of a button [27]–[29].

The potential benefits of these developments are considerable and multidimensional. It is important to note however that potential benefits do not equate with those that have been empirically demonstrated. More importantly, we must not ignore the potential risks associated with the implementation of ICT in complex environments such as health care services.

## Recognising the Risk of Harm

As noted above, a number of governments around the world are currently engaging in truly epic programmes to roll out eHealth interventions as quickly as possible throughout the health care sector [32]. However, it is of concern that, in this rush, relatively little time, thought, or resources have been devoted to assessing the potential risks associated with eHealth interventions [11]–[13],[33]–[36].

eHealth may compromise patient safety in a number of ways. Take the example of poorly developed computer decision support functionality, which can result in the issuing of erroneous prescribing support and advice, as was demonstrated by Fernando et al. in their assessment of primary care prescribing software [33].

Another cause for concern is the roll-out of applications that allow round-the-clock, multiple access points to patient data through health portals such as Google Health, Microsoft's HealthVault, and the National Health Service's HealthSpace [27]–[29]. These applications have been designed to allow users to read, amend, and share their medical records with others, be they health care professionals, support groups, caregivers, family, friends, and/or other patients. But what mechanisms are in place to prevent cyber criminals from accessing or stealing sensitive data [34]–[36]? How will these and other eHealth applications be suitably evaluated so they are (and remain) secure portals for storing and exchanging potentially sensitive information [18]?

Box 2 details some high profile examples of ways in which eHealth deployments may have compromised patient safety and/or confidentiality.

**Box 2. Examples of problems associated with eHealth projects**

- Large investments in eHealth may, by diverting resources result in a shortfall in funding for basic infrastructure, equipment, and staffing elsewhere in the system. For example, some community health centres in South Asia do not have the facilities or the surgical staff to carry out such basic procedures as a caesarean section, so investing in developments such as telemedicine, which are only likely to be accessible to a minority, would exacerbate the digital divide and existing health inequities [11].
- eHealth applications that are inappropriately specified, have functional errors, are unreliable, user-unfriendly, or in a poorly prepared or supported environment can put patients' and the health service at risk. For example: (i) patients from Michigan were wrongly coded as being dead on medical bills; (ii) increased workloads on clinical users can in turn decrease in the quality of patient care; and (iii) delays in answering emergency calls due to problems with emergency dispatching systems may lead to delays in emergency treatment [12].
- An unexpected increase in mortality was observed after the implementation of a commercially sold computerised physician order entry system; this increase may have been due to “system integration failure” and/or “human-machine interface flaws” [13].
- Installation of “Trojan horse” software on end-user computers can capture: key stroke information, files stored on hard drives and Microsoft Outlook e-Mail files, all of which allow cyber criminals to steal electronic health records [36].

## Implications for Evaluation

In order to maximise benefits and minimise risks, eHealth interventions need to be subject to the same independent scrutiny as any other health care intervention prior to implementation, i.e., they need to be suitably evaluated. Such evaluations need to begin with a clear description of a problem or need; for example, the need to improve access to health care information for both professionals and patients. A chain of reasoning then must take place that leads from the statement of the problem or need to the formulation of a possible solution (see Box 3) [37]. If any part of that chain is missing, it is highly probable that a poor-quality solution, or even a wrong solution, will be developed. As noted above, in the context of health care the implications of such failures may be particularly profound [11]–[13], but an evaluation programme capable of evaluating each part of this chain will help ensure that the right solution is developed and delivered for the need, whilst also recognising the importance of local contextual considerations.

**Box 3. Suggested seven steps from statement of a problem to definition of a solution [37]**

1. **Drivers:** Clearly articulate why change is needed (e.g., problems posed by paper-based records in allowing ready multiple user access to patient records when needed).
2. **Vision:** Realistically define possible responses to those drivers, i.e., what the revised model of delivering care will look like (e.g., patient records will be readily accessible from anywhere within the health care institution/setting).
3. **Goals:** Explain how a project will move toward realising this vision (e.g., electronic health records will be deployed through a web-based secure network).
4. **Business objectives:** Define how success will be measured (e.g., health care professionals, patients, their caregivers, and the public at large will have access to electronic health information over a secure network, from anywhere at any time, within x years at y cost). It is important that these timelines and costs are realistic.
5. **Business requirements:** Define the detailed capabilities that will be needed in order to achieve these business objectives (e.g., detailed technical specifications; access to authority; social [i.e. behavioural and organisational] changes).
6. **Design:** Propose possible solutions to meet the need and these requirements (e.g., commissioning of a custom-made Web-based electronic health record system).
7. **Solution:** Develop and implement the solution and assess whether the problems have been resolved and the anticipated benefits realised within the proposed time scale and allocated budget.

Within biomedicine, randomised controlled trials (RCTs) are often seen as the “gold standard” methodological approach, rightly so because of their unique ability to control for the impact of known and unknown confounding factors [16]. But whilst RCTs and similar experimental design methods may be appropriate for studying interventions under controlled clinical conditions, these design methodologies alone are often less well suited to evaluate the impact of eHealth interventions in a complex environment or to study the effect they have on the delivery of care [16],[17]. The main reason for this deficiency is that studies adopting an experimental design approach fail to take sufficient account of the contextual considerations, which play a major role in the success or failure of the intervention being studied. It is therefore often difficult simply to generalise from results obtained using RCTs when studying complex interventions such as eHealth technologies [16],[17],[20].

**Figure 1:**
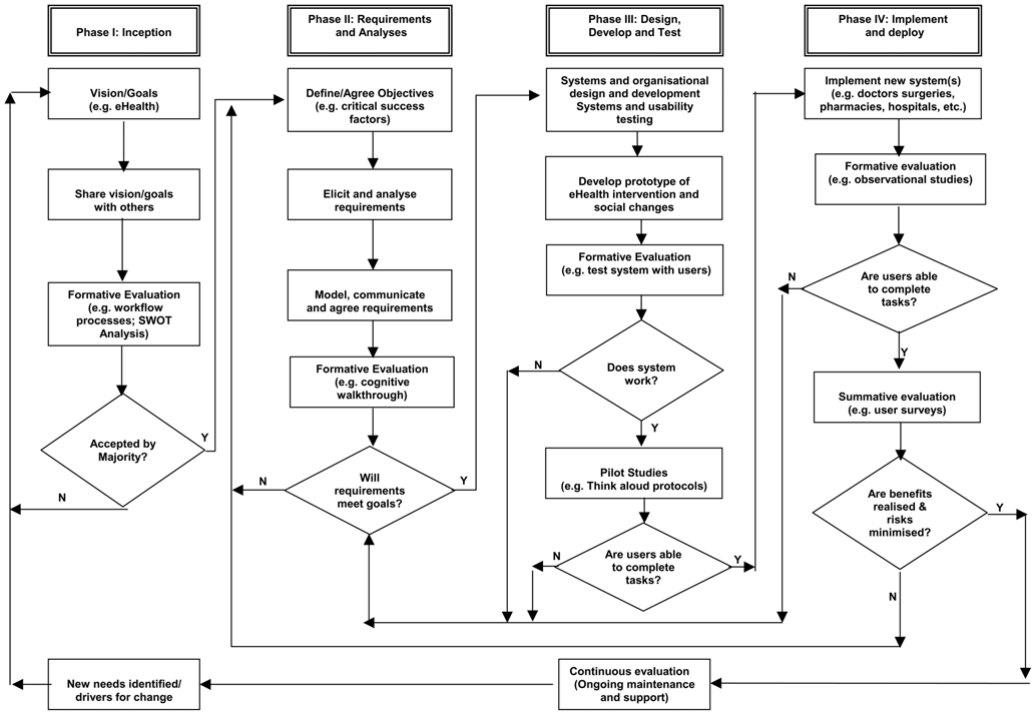
Schematic for simplified evaluation process.

In light of these considerations, and the current dilemma regarding investing in eHealth [17], we propose an alternative more comprehensive overall evaluation approach, one that encourages a multifaceted, multidisciplined approach and facilitates continuous systematic evaluations throughout the lifecycle of an eHealth intervention. The proposed approach takes into account sociotechnical and contextual considerations [38] and is capable of ensuring that each part of the chain of reasoning is adhered to [37]. Figure 1 depicts our suggested evaluation approach.

The overall aim of this model is to maximise the benefits while minimising any risks associated with the eHealth intervention. This balance is achieved by iterative formative evaluations at four key stages of the eHealth intervention's lifecycle [20]. This model has the additional advantage of providing a means to understand the implementation process [17].

From the very beginning of an idea, for example, the replacement of paper-based records with electronic health records, it is important to be able to describe the vision, i.e., what will the new health service look like, including identifying in as much detail as possible measures of success/benefits, potential risk/costs, main stakeholders, and potential social changes [31]. The aim here is to gauge whether or not the idea has any perceived merit and, if so, which areas, if any, may need to be reconsidered. In this respect it is important that design teams take a multifaceted and multidisciplined approach to documenting the complex relationships between the political, social, organisational, and technical worlds. At some point, the rich picture of the real world needs to be developed into a conceptual model so that stakeholders can reflect critically on the drivers, vision, and goals of the project and agree whether or not such a programme of change is appropriate and feasible (see Figure 1: Phase I).

Only once the initial idea has been debated and accepted by the majority of key stakeholders should one proceed to the next stage, i.e., requirements elicitation and analyses. In this stage, design teams need to gain a thorough understanding of the stakeholders' needs, concerns, values, and beliefs, and define (as far as possible) what the eventual system will be expected to provide. It is important that this initial elicitation stage goes beyond functional and technical requirements and considers, for example, accessibility, acceptability, and affordability issues. Formative iterative evaluations using simple prototypes of the eHealth intervention may be useful at this stage to assist with the communicating of ideas, building a common understanding, agreeing to objectives, and securing stakeholder buy-in (see Figure 1: Phase II).

The third phase of the project involves the design, development, and testing of a system, including assessment and adoption of the social changes necessary to make the new system work [31]. Once a working model of the system is available, empirical evaluations can be completed, which could include the collection of quantitative and/or qualitative data, depending on the goals and scope of the study and the stage of development [20]. This stage of the evaluation process is likely to highlight any design faults and/or training needs. Therefore it is extremely important to take this opportunity to refine the design and/or address training needs before the system is rolled out to further sites (see Figure 1: Phase III).

The final stage is to implement and deploy the (working and accepted) system across the health sector, and in the process to undertake a series of formative evaluations of the system in operation under normal/everyday conditions. A summative evaluation should also be conducted to verify whether or not the new system meets the purpose (requirements) for which it was designed, whether or not the associated benefits have been realised, and whether or not there are any risks to patients and/or the health care system. At this stage, it is important to demonstrate that the original need has been satisfactorily met, opportunities to improve the system are highlighted, and “drivers for change” identified, whereby the cycle begins again (see Figure 1: Phase IV).

## Conclusions

eHealth interventions have considerable potential to transform the health sector, hopefully for the better. As with any other intervention, however, the risk of harm exists, so policymakers, commissioners, clinicians, and patients alike need to remain aware of this possibility. If we are to maximise the benefits associated with eHealth interventions whilst minimising risks, we must be able simultaneously to evaluate eHealth interventions while they are being designed, developed, and deployed [20].

In this article, we have proposed a novel approach to evaluation that we believe addresses this need, while facilitating an evaluation of events leading up to the new system and continuing long after it has been implemented. Such systematic evaluation matters, because at the end of the day it must be hard scientific evidence that informs key policy decisions, rather than, as is currently so often the case, industry lobbying, political expediency, or enthusiasm to implement technology simply because it exists.

## Abbreviations

ICT: information communication technology
RCT: randomised controlled trial
